# Letter to the Editor for the article ‘Prognosis of uterine and extrauterine low-grade endometrial stromal sarcoma: an observational cohort study’

**DOI:** 10.1097/JS9.0000000000001710

**Published:** 2024-05-29

**Authors:** Weining Fan, Lexin Wang, Yaoqin Xu, Hao Chi

**Affiliations:** a General Hospital of Ningxia Medical University; b Ningxia Medical University, Yinchuan, Ningxia; c Clinical Medical College, Southwest Medical University, Luzhou, People’s Republic of China

*Dear Editor,*

The study conducted by Dai *et al*.^1^ in the ‘*International Journal of Surgery*’ has garnered significant interest, and we commend their innovative findings. Low-grade endometrial stromal sarcoma (LGESS) is a rare malignant tumor, and this research provides new insights into the prognosis of LGESS, filling a knowledge gap in the field. The authors note that the clinical characteristics of extrauterine LGESS are similar to those of uterine LGESS. Additionally, they found that ovarian preservation significantly increases the risk of LGESS recurrence^1^. These findings hold potential value for the diagnosis and treatment of patients and lay a foundation for further research and clinical practice. However, there are several noteworthy issues with this study.

Firstly, the study included a total of 143 primary LGESS patients and 56 recurrent LGESS patients. However, it may still be insufficient to draw comprehensive and reliable conclusions^2^. Additionally, an insufficient sample size can potentially threaten the accuracy of the data. Therefore, the authors need to include more samples for subsequent analysis.

Secondly, there is insufficient control of potential confounding factors in the study. While the authors mention some potential confounding factors such as ovarian preservation, other factors such as patient age, BMI, family history, etc., which are not adequately considered, may lead to bias in the study results^3^. Therefore, a more comprehensive investigation and control of these factors may help reduce the influence of confounding factors.

Third, the limitations of the study design are also worth noting. Although this study provides important observational data, as an observational study, it can only provide correlations and cannot establish causality. This means that although some associations can be observed, it cannot be determined whether they are causal relationships. Additionally, observational studies are prone to observation bias and subjective judgments by researchers, which may lead to problems in result interpretation. Future research could employ more rigorous study designs, such as randomized controlled trials, to validate these observational findings and better understand the pathogenesis and treatment effects of LGESS^4^.

Finally, I would like to express my gratitude to the author for the conclusion of the article and their contribution to clinical treatment.

## Ethical approval

Not applicable.

## Consent

Not applicable.

## Sources of funding

This work was supported by Ningxia Natural Science Foundation (2022AAC03573).

## Author contribution

W.F., L.W., Y.X., and H.C.: conceptualization, methodology, validation, formal analysis, investigation, resources, data curation, writing – original draft, writing – review and editing, supervision, and project administration.

## Conflicts of interest disclosure

There are no conflicts of interest.

## Research registration unique identifying number (UIN)

Not applicable.

## Guarantor

Weining Fan, Lexin Wang, Yaoqin Xu, and Hao Chi.

## Data availability statement

Data sharing is not applicable to this article.

## Provenance and peer review

Not applicable.

## Assistance with the study

Not applicable.
